# The presentation and management of post-partum choriocarcinoma

**DOI:** 10.1038/sj.bjc.6690244

**Published:** 1999-03

**Authors:** L S Dobson, A M Gillespie, R E Coleman, B W Hancock

**Affiliations:** Trophoblastic Disease Screening and Treatment Centre, YCR Section of Clinical Oncology, Whitham Road, Sheffield, S10 2SJ, UK

**Keywords:** post-partum, chemotherapy

## Abstract

Post-partum choriocarcinoma is a rare complication of pregnancy. We have analysed a series of nine consecutive patients presenting with choriocarcinoma after a full-term non-molar pregnancy. All patients were managed at the Supraregional Trophoblastic Disease Screening and Treatment Centre at Weston Park Hospital, Sheffield between 1987 and 1996. All presented with persistent primary or secondary post-partum haemorrhage. Treatment with multiagent chemotherapy (initially methotrexate, dactinomycin and etoposide) was successful in all cases. Early diagnosis is important because this rare condition is potentially curable with appropriate chemotherapy. © 1999 Cancer Research Campaign


*British Journal of Cancer* (1999) **79**(9/10), 1531–1533
© 1999 Cancer Research Campaign
Article no. bjoc.1998.0244

### The presentation and management of post-partum choriocarcinoma

**LS Dobson, AM Gillespie, RE Coleman and BW Hancock**

Trophoblastic Disease Screening and Treatment Centre, YCR Section of Clinical Oncology, Weston Park Hospital, Whitham Road, Sheffield S10 2SJ, UK

**Summary** Post-partum choriocarcinoma is a rare complication of pregnancy. We have analysed a series of nine consecutive patients presenting with choriocarcinoma after a full-term non-molar pregnancy. All patients were managed at the Supraregional Trophoblastic Disease Screening and Treatment Centre at Weston Park Hospital, Sheffield between 1987 and 1996. All presented with persistent primary or secondary post-partum haemorrhage. Treatment with multiagent chemotherapy (initially methotrexate, dactinomycin and etoposide) was successful in all cases. Early diagnosis is important because this rare condition is potentially curable with appropriate chemotherapy.

**Keywords:** post-partum; chemotherapy

Gestational trophoblastic disease (GTD) is a term that incorporates a rare but important spectrum of disorders that may complicate pregnancy. GTD is most commonly associated with an abnormal conception (either a complete or partial hydatidiform mole or more rarely choriocarcinoma) but it may follow a normal delivery or non-molar abortion. Choriocarcinoma following a live birth is said to have an incidence of 1 in 50 000 births (Bagshawe et al, 1986) and to be associated with a poor prognosis (Bagshaw, 1976; Tidy et al, 1995; Bower et al, 1997). We have evaluated our experience in the management of post-partum choriocarcinoma by undertaking a retrospective analysis of all patients registered and treated for persistent trophoblastic disease (PTD) between 1987 and 1996 at Weston Park Hospital, specifically identifying those patients with post-partum choriocarcinoma.

### METHODS

The Weston Park Hospital database of gestational trophoblastic tumours was used to identify all women treated for histologically confirmed choriocarcinoma. Patients who presented following full-term non-molar pregnancy had their case notes studied to identify patient demographics, obstetric history, presentation, treatment and outcome.

Our current management policy on referral of patients with PTD is to review and confirm the histological diagnosis of choriocarcinoma: the patient undergoes a prognostic risk assessment including full physical examination, imaging [ultrasound pelvis and abdomen, computed tomography (CT) thorax, chest X-ray] and serum β human chorionic gonadotrophin (βhCG) evaluation prior to initiation of treatment as determined by our modified Charing Cross prognostic scoring system (Sheridan et al 1993). Those patients at high risk of central nervous system involvement also undergo a CT scan of the head and a lumbar puncture (provided there is no evidence of increased intra-cranial pressure) to determine the cerebro-spinal fluid:serum βhCG ratio.

*Received 29 September 1998*
*Accepted 7 October 1998*

*Correspondence to:* BW Hancock

### RESULTS

Between January 1987 and December 1996, 225 women with PTD were treated at Weston Park Hospital. Thirty-one patients received high risk chemotherapy. In nine patients (29%) the antecedent pregnancy was a full-term non-molar pregnancy. In this group, the median age was 29 years (range 22–37), and eight of nine women were multiparous. There was no sexual preponderance in the antecedent pregnancy (four male, five female).

The time interval from antecedent pregnancy to onset of symptoms ranged from 0 to 12 weeks (median 2 weeks). All nine patients presented with abnormal vaginal bleeding. In four cases this was persistent from the time of delivery, while in the other five cases the post-partum haemorrhage commenced at a median of 5 weeks after delivery (range 2–12 weeks). Only two women experienced lower abdominal pain.

The interval between the antecedent pregnancy and registration for treatment ranged from 5–25 weeks (median 10 weeks). In one case 24 weeks elapsed before referral. The patient underwent three uterine evacuations and only during the final operation was tissue sent for histological analysis. In a second case, vaginal bleeding persisted for 3 months from the time of birth, and during this period the patient received norethisterone and finally presented 17 weeks post-partum with dyspnoea secondary to multiple pulmonary metastases. Another case presented at 16 weeks post-partum. She started bleeding 5 weeks after delivery and underwent a uterine evacuation at week 7. Despite the histology being reported as suspicious of choriocarcinoma, the patient went on to receive three courses of intramuscular medroxyprogesterone acetate before the appropriate referral was made at 16 weeks post-partum.

By the time of referral seven of nine patients had metastatic disease. However, these metastases resulted in symptoms in only two cases: one patient experienced 3 weeks of progressive dyspnoea; one patient was diagnosed after the death of her

**Table 1** Summary of findings in nine patients with post-partum choriocarcinoma

| Patient | A | B | C | D | E | F | G | H | I |
|---|---|---|---|---|---|---|---|---|---|
| Onset of PV bleeding (weeks post-partum) | 12 | Primary PPH | Primary PPH | 3 | 2 | 6 | 5 | Primary PPH | Primary PPH |
| Diagnosis (weeks post-partum) | 24 | 7 | 17 | 9 | 7 | 16 | 10 | 7 | 19 |
| Interval to diagnosis (weeks) | 12 | 7 | 17 | 6 | 5 | 10 | 5 | 7 | 19 |
| Number of metastases | Multiple | Multiple | Multiple | Multiple | Single | Multiple | Multiple | Nil | Nil |
| Site of metastases | Lungs | Lungs | Lungs | Lungs CNS | Lungs | Lungs | Lungs | Nil | Nil |
| Serum βhCG at diagnosis (iu $l^{-1}$) | 258 500 | 238 500 | 265 000 | 286 500 | 202 500 | 665 000 | 808 500 | 800 000 | 93 050 |
| Treatment | MAE (10) | MAE (8) | MAE (8) CEC (3) | MAE (9) IT MTX | MAE (8) | MAE (7) | MAE (7) | EMACO (2) MAE (6) | MAE (7) |
| Survival | AWNED | AWNED | AWNED | AWNED | AWNED | AWNED | AWNED | AWNED | AWNED |
| Duration of follow-up (years) | 5 | 4 | 2 | 2 | 7 | 6 | 6 | 5 | 5 |

AWNED, alive and well, no evidence of disease; βhCG, human chorionic gonadotrophin; CEC, cyclophosphamide/etoposide/cisplatin; EMA-CO, etoposide/methotrexate/actinomycin D (dactinomycin) – cyclophosphamide/Oncovin (vincristine); IT, intrathecal; MAE, methotrexate/actinomycin D (dactinomycin)/etoposide; MTX, methotrexate; PPH, post-partum haemorrhage; PV, per vaginum.

newborn infant at 4 weeks of age due to choriocarcinoma (Picton et al, 1995). There was no relationship between the time to presentation or referral and the presence of metastases.

All nine patients who presented had high-risk disease, with a median risk score of 14 (range 10–23). Of note is that the median level of βhCG at the time of referral was 286 500 iu $l^{-1}$ (range 93 050–808 500 iu $l^{-1}$).

All patients received 'high-risk' chemotherapy with MAE. This is an alternating intravenous regimen of methotrexate 300 mg $m^{-2}$, with folinic acid rescue, followed by a 7-day rest period and then dactinomycin (actinomycin D) 0.5 mg $day^{-1}$ and 3 days of etoposide 150 mg $m^{-2}$ $day^{-1}$. Treatment was continued until the βhCG was less than 2 iu $l^{-1}$ and then continued for a further 8 weeks. In addition, one patient received high-dose intravenous Methotrexate (1 g $m^{-2}$) and intrathecal methotrexate (12.5 mg) for confirmed cerebral metastases. Another required conversion to second-line therapy for refractory disease after eight cycles of treatment. She had a further three courses of chemotherapy with CEC (cisplatin 25 mg $m^{-2}$ daily for 3 days, etoposide 100 mg $m^{-2}$ daily for 3 days and cyclophosphamide 600 mg $m^{-2}$ for 1 day only, repeated at 7- to 10-day intervals). One patient initially received two courses of EMA-CO [etoposide, methotrexate, dactinomycin (actinomycin D), cyclophosphamide, vincristine (Oncovin) (Bower et al, 1997)] chemotherapy in Australia before returning to this country where she completed a further six courses of MAE chemotherapy uneventfully.

Chemotherapy was generally well tolerated. The median number of cycles of chemotherapy received was eight (range six to 10). Only one patient had a treatment cycle delayed for more than 3 weeks. Six patients exhibited grade III or IV neutropenia and in three of these neutropenic sepsis resulted. Two patients were given growth factor (lenograstim) support and two patients had a dose reduction. Nausea and vomiting were minimal and all patients exhibited temporary alopecia.

All patients achieved a complete response and remain disease-free at a median of 5 years (range 2 to 7 years). There was no treatment- or disease-related mortality seen in this group of patients. All are followed up regularly with urine hCG levels for life.

Future fertility in this patient group does not appear to be affected. Five women have subsequently had further pregnancies without complication. Three women have no desire to become pregnant including one who underwent a hysterectomy during chemotherapy as her family was complete. The ninth patient has not had a further pregnancy and her future fertility wishes are not known. None of our patients has had a subsequent pregnancy complicated by choriocarcinoma or a hydatidiform mole.

### DISCUSSION

Post-partum choriocarcinoma is a very rare complication of pregnancy with a reported UK incidence of 1 in 50 000 live births (Bagshawe et al, 1986). The rarity of this condition is reflected in our own experience. During the study time period we received nearly 4000 registrations with GTD and managed 225 cases of PTD of whom only nine presented with post-partum choriocarcinoma.

In our experience, results with multiagent systemic chemotherapy are excellent and all our patients are alive and well with no evidence of disease. The Charing Cross Hospital team, however, has reported from their major experience over 25 years that choriocarcinoma following a term non-molar pregnancy is an adverse factor, in both univariate and multivariate analyses. By reviewing their 272 cases requiring high-risk multiagent (EMA/CO) chemotherapy they found that 99 cases (36%) of

*© Cancer Research Campaign 1999*

persistent trophoblastic disease followed term pregnancy. Although our patients are consecutive and recently treated it is possible that with the number being much smaller (nine patients, 29% of all those requiring high risk chemotherapy) too optimistic a view was obtained.

Of concern, however, is the time interval/delay between onset of symptoms and diagnosis with subsequent referral for treatment. Whilst there are of course a number of possible explanations for post-partum haemorrhage, many of our patients could have been referred sooner. Simply sending curettings for histological analysis, performing a βhCG analysis or having some index of suspicion if abnormal bleeding per vaginum persists despite therapeutic intervention would have led to earlier diagnosis in some cases. Delays in diagnosis certainly resulted in increased patient morbidity from choriocarcinoma and may have resulted in more frequent usage of higher dose multiagent chemotherapy (seven of nine patients had metastatic disease on referral with a median βhCG of more than 250 000 iu $l^{-1}$).

In conclusion, the important message is that although this condition is extremely uncommon it is potentially treatable and an early diagnosis is important.

### REFERENCES

Bagshawe KD, Dent J, Webb J (1986) Hydatidiform mole in England and Wales in 1973–1983. *Lancet* **i**: 673–677

Bagshawe KD (1976) Risk and prognostic factors in trophoblastic neoplasia. *Cancer* **38**: 1373–1385

Bower M, Newlands ES, Holden L, Short D, Brock C, Rustin GJS, Begent RHJ and Bagshawe KD (1997) EMA/CO for high risk gestational trophoblastic tumours: results from a cohort of 272 patients. *J Clin Oncol* **15**: 2636–2643

Picton SV, Bose-Haider B, Lendon M, Hancock BW and Campbell RHA (1995) Simultaneous choriocarcinoma in mother and newborn infant. *Med Pediatr Oncol* **25**: 475–478

Sheridan E, Hancock BW, Smith SC, Doreen MS, Neal FE, Pennington GW and Millar DR (1993) Gestational trophoblastic disease: experience of the Sheffield (United Kingdom) supraregional screening and treatment service. *Int J Oncol* **3**: 149–155

Tidy JA, Rustin GJS, Newlands ES, Foskett M, Fuller S, Short D and Rowden P (1995) Presentation and management of choriocarcinoma after nonmolar pregnancy. *Br J Obstet Gynaecol* **102**: 715–719

© Cancer Research Campaign 1999
